# A Trade-Off Between Sporangia Size and Number Exists in the Potato Late Blight Pathogen *Phytophthora infestans*, and Is Not Altered by Biotic and Abiotic Factors

**DOI:** 10.3389/fpls.2018.01841

**Published:** 2018-12-19

**Authors:** Nicolas Mariette, Alexander Kröner, Romain Mabon, Josselin Montarry, Bruno Marquer, Roselyne Corbière, Annabelle Androdias, Didier Andrivon

**Affiliations:** INRA, UMR1349 Institute for Genetics, Environment and Plant Protection, Le Rheu, France

**Keywords:** fitness, life-history traits, plant pathogen, reproductive strategies, *Solanum tuberosum*

## Abstract

The negative relationship between offspring size and number is a classic example of trade-off between life-history traits, reported many times in animal and plant species. Here, we wanted to ascertain whether such a trade-off occurred in the oomycete *Phytophthora infestans*, and whether it was impacted by biotic and abiotic factors. We thus conducted three infection experiments under controlled conditions and measured the number and the size of sporangia (asexual propagules) produced on potato by different *P. infestans* isolates. In all experiments, we observed a negative relationship between sporangia size and number, demonstrating the existence of a trade-off. Moreover, although the potato host cultivar, temperature and host of origin (tomato or potato) all affected sporangia number, sporangia size or both, none of these biotic and abiotic factors did change the trade-off. Therefore, the trade-off between sporangia size and number could maintain the polyphenism for these traits in *P. infestans* populations, and favors the coexistence of distinct reproductive strategies within this species. Our results emphasize the relevance to focus on the relationship between offspring size and number in other fungal plant pathogens, as well as to study the impact of offspring size on fitness-linked traits (virulence and disease lesion development) in these organisms.

## Introduction

The widely accepted trade-off concept states that an organism cannot simultaneously maximize all traits involved in fitness, owing to dilemmas in resource allocation (Law, 1979). According to this theory, any beneficial change in one trait will unavoidably have a detrimental effect on another trait (Anderson and May, 1982; Stearns, 1989). The net result is a negative correlation between traits that mobilize the same resources. The trade-off concept has a prominent importance in evolutionary biology, as it can explain how the adaptation of populations to new environments can be constrained and how life-history strategies can evolve (Rausher, 1984; Roff, 1992). Trade-offs between fitness components are of central importance in plant pathogens, as they could limit their evolutionary potential (Lannou, 2012; Laine and Barres, 2013) and may thus be exploited to engineer durable control strategies (Quenouille et al., 2013; Brown, 2015).

Since they are key to determining fitness, trade-offs involving reproductive traits, especially the one between offspring size and number, have received the most attention in the scientific literature (Stearns, 1989; Roff, 2002; Begon et al., 2006). The trade-off between offspring size and offspring number is based on the hypothesis that, for a given total reproductive investment, if most resources are devoted to the production of many offspring individuals, each of them will have limited lower share of the reproductive investment (Begon et al., 2006). If this trade off exists (that is, if resources available for reproduction are constrained), reproductive strategies should range along a continuum, from the production of few large offsprings to the production of many small ones (Timi et al., 2005). This size-number trade-off was first advocated by Lack (1947) with experiments on clutch-size in birds, and has been subsequently observed in many animals (Charnov and Ernest, 2006; Walker et al., 2008) and plants (Werner and Platt, 1976; Jakobsson and Eriksson, 2000; Linkies et al., 2010).

The size-number trade-off could also be found in plant parasites, especially oomycetes and fungi. For such sporulating organisms (with spores as asexual propagules), producing large spores should indeed be more energetically costly than producing small spores, but may increase spore viability or infectious ability. Although trade-offs involving spore production on one hand and other life-history traits such as latent period (Pariaud et al., 2013), virulence (Thrall and Burdon, 2003; Montarry et al., 2010) or transmission success rate (Pasco et al., 2016) on the other hand, were observed in various plant pathogens, very few studies have investigated the existence of a trade-off between spore size and number (Delmotte et al., 2014). Fortunately, the advent of new techniques and equipments for counting and measuring thin particles like spores now make the exploration of such a trade-off possible.

As in all pathogenic interactions, the development of a disease caused by a plant pathogen is strongly influenced by both abiotic environment and host genotype (Laine, 2007; Wolinska and King, 2009; Clément et al., 2010; Delmotte et al., 2014). As a consequence, these factors can also have an impact on the shape and even the direction of the correlation between life-history traits in pathogens. Hence, fitness costs can differ among environments (e.g., temperature) as observed in the fungal pathogen *Podosphaera plantaginis* (Laine, 2008). Likewise, some studies reported shifts of life-history correlations in plant pathogens, depending on the cultivars tested (Huang et al., 2010; Susi and Laine, 2013). These data thus stress that it is crucial to take into account sufficient variation in both the host and the parasite, by measuring life-history traits in different environments, to draw conclusions on the status of a life-history trade-off in a plant pathogen.

Here, we aimed to investigate the existence of a trade-off between offspring number and size in the oomycete *Phytophthora infestans*, and the impact of some biotic and abiotic factors on this eventual trade-off. Infamous for having triggered the Irish Great Famine in the 1840s, *P. infestans* is the causing agent of late blight in solanaceous species and is responsible for significant losses in both potato and tomato crops (Fry et al., 2015). This pathogen is particularly suitable for exploring trade-offs, as it can be isolated and maintained as axenic cultures on artificial media whereas miniature biotests performed in controlled conditions can be used for the assessment of life-history traits of the pathogen, particularly those linked to spore formation (Montarry et al., 2010). We thus conducted three experiments under controlled conditions in which we measured the size and number of sporangia (*i.e.*, asexual spores) produced by *P. infestans* isolates after artificial inoculations on potato leaflets. In each experiment, we changed one factor [host resistance level, temperature, or the host of origin (tomato or potato)] which could impact the trade-off. We measured the effect of these factors on both sporangia size and number, analyzed the relationships between these two traits and compared them within each set of conditions.

## Materials and Methods

We conducted three inoculation experiments of *P. infestans* isolates on detached potato leaflets in which we measured the size and the number of sporangia produced by the pathogen. Different biotic and abiotic parameters – host resistance level, temperature and host of origin (tomato or potato) – were modified in these experiments. The experimental protocols used for these experiments were mostly similar but contained some slight differences (Table 1).

**Table 1:** Protocol specificities applied for each experiment lead during the study.

	Experiment 1	Experiment 2	Experiment 3
Inoculum concentration	5 × 10^4^ sporangia⋅ mL^-1^	5 × 10^4^ sporangia⋅ mL^-1^	3 × 10^4^ sporangia⋅ mL^-1^
Potato cultivars	• Bintje	Bintje	Bintje
	• Robijn		
	• Möwe		
Thermal regimes	15/18°C (day/night)	• 10°C	15/18°C (day/night)
		• 14°C	
		• 18°C	
Time of the sporangia collection	3 days after latent period	3 days after latent period	5 days after inoculation
No. independent replicates	1	2	2


### Isolate Collections

Single-lesion isolates were established and maintained as axenic cultures on pea agar medium as previously described (e.g., Montarry et al., 2010). Briefly, each single-lesion isolate was obtained by placing a fragment of infected leaf tissue on tuber slices of the susceptible potato cultivar Bintje. After incubation at 15°C in growth chambers for four to eight days, pure cultures were established by transferring the hyphal tips growing through the slices to sterile pea agar and subsequently maintained at 15°C in darkness by serial transfers on fresh pea agar.

In the first experiment, we used 119 isolates sampled in commercial potato fields of western France during late blight epidemics of 2014 (Mariette, 2016). All isolates were tested on three potato cultivars: Bintje, Robijn, and Möwe. These cultivars do not bear race-specific resistance genes but differ in levels and components of partial resistance to late blight: Bintje is the reference susceptible cultivar to *P. infestans* commonly used in European laboratories, whereas the partially resistant Robijn and Möwe notably reduce spore production of the pathogen (Clément et al., 2010). In the second experiment, 16 western French isolates sampled in 2013 were tested on the potato cultivar Bintje under three thermal regimes: 10, 14, and 18°C. This temperature range was chosen because it covers the biological activity of *P. infestans* (Maziero et al., 2009). The third experiment included 21 isolates sampled either on potato (11) or tomato (10), in French and Algerian crops from 2013 to 2015, and were tested on the potato cv. Bintje.

### Plant Material

Three potato cultivars (Bintje, Robijn, and Möwe) and one tomato cultivar (Marmande) were used. Certified seed tubers of the potato cultivars were obtained from the INRA Biological Resource Center BrACySol (Ploudaniel, France), whereas commercial standard seed of the tomato cultivar was used.

Plants were grown in pots filled with 1:1:1 sand-peat-compost mixture placed in a glasshouse regulated at 15–20°C (night/day temperatures) with 16 h of photoperiod. Once a week, plants were watered with a nutrient solution (Hakaphos; NPK 15/10/15). For the inoculum preparation and life-history traits measurements, leaflets of similar size were picked from the median area of 6- to 8-week-old plants.

### Inoculum Preparation

Prior the assessment of their life-history traits and in order to restore pathogenicity possibly lost in axenic cultures (Jinks and Grindle, 1963), *P. infestans* isolates were first inoculated onto detached leaflets of the original host species: the potato cv. Bintje and the tomato cv. Marmande for potato and tomato isolates, respectively. For this purpose, droplets of sporangial suspensions, prepared from 3- to 4-week-old pea agar cultures by flooding with sterile water and scrapping the colony surface, were deposited on the lower (abaxial) side of detached leaflets kept on the empty lids of inverted Petri dishes containing 10 g L^-1^ water agar and acting as humid chambers. After 7 days of incubation at 18/15°C (day/night temperature) and 16-h day length, newly formed sporangia were collected from infected leaflets by gently shaking in sterile water. Sporangia were then counted using a hemocytometer, adjusted to a final concentration of 3 × 10^4^ or 5 × 10^4^ sporangia mL^-1^, depending on the experiment (Table 1), and then kept at 4°C for 2 h to promote zoospore release.

### Inoculation

Sporangial suspensions were inoculated onto a potato leaflet, except in the second experiment where three leaflets of potato cv. Bintje were inoculated due to the three thermal regimes tested (Table 1). To this end, leaflets were placed, abaxial face up, onto the empty lids of inverted Petri dishes containing 10 g L^-1^ water agar. A 20-μL droplet of the prepared sporangial suspension was then deposited on the center of the leaflet. The Petri dishes were kept in clear boxes and incubated in climate chambers regulated at thermal conditions desired (Table 1) with a 16 h photoperiod.

### Life-History Traits Measurements

Three days after the formation of the first sporangia – checked by daily observations under a magnifying glass – or 5 days after inoculation (depending on the experiment, Table 1), newly formed sporangia were washed from each leaflet in 10 mL Isoton II (saline buffer; Beckman Coulter, Villepinte, France). Suspensions were kept in glass tubes at -20°C until the counting of sporangia number (SN) produced using a Coulter Z2 counter (Beckman Coulter) equipped with a 100-μm aperture tube. Sporangia size (SS) was determined under a microscope fitted with a camera and using the image analysis software Histolab^®^ v8.1.0 (Microvision Instruments, Evry, France). For this purpose, drops of the sporangial suspension were placed under the microscope and we used the camera measurement software to determine length and width of 100 randomly chosen sporangia. These measures allowed to calculate sporangia volumes, assuming a revolution ellipsoid shape for each sporangium (Philibert et al., 2011) by applying the standard equation $\frac{4\text{π}}{3}*\frac{length}{2}*{\left(\frac{width}{2}\right)}^{2}.$

### Statistical Analyses

All analyses were performed using the statistical software R v. 3.4.4 (R Core Team, 2017) and the significance threshold was fixed at α = 0.05. We first compared SS and SN of *P. infestans* isolates tested on different potato cultivars (experiment 1) and at different temperatures (experiment 2) using analysis of variance (ANOVA) followed by Tukey’s honestly significant difference *post hoc* comparisons. For the comparisons between isolates sampled on potato and tomato (experiment 3), we applied a Student *t*-test. When necessary, SS and SN were log-transformed to achieve assumptions of homoscedasticity and normality. For the three tested modalities (potato cultivar, temperature and host of origin), each *P. infestans* isolate was regarded as a biological repetition. Then, we investigated the relationship between SS and SN for each modality tested in the three experiments using Pearson correlations and linear regressions (where SS and SN were log-transformed). To test whether slopes of regression lines differed between modalities within each experiment, variation in SS was analyzed by mean of analysis of covariance (ANCOVA) with SN as the covariate and host cultivar, temperature or host of origin as the factor. In the first experiment implying 119 isolates, phenotypic measurements were made only once on each of them as the large number of tested individuals allowed a good assessment of significance. In the case of experiments 2 and 3, we compared data obtained from the two independent replicates and, as results showing no significant differences (*i.e.*, the criterion was statistically fulfilled or rejected), data from both experiments were pooled, reanalyzed and represented graphically.

## Results

### Impact of Biotic and Abiotic Factors on *P. infestans* Life-History Traits

In experiment 1, potato cultivar had a significant effect on SS of *P. infestans* isolates (ANOVA, *F*_2,352_ = 3.58, *P* = 0.029), with bigger sporangia produced on Bintje than on Robijn as revealed by Tukey *post hoc* tests (Table 2). Likewise, a significant effect of potato cultivar on SN was detected (ANOVA, *F*_2,352_ = 158.48, *P* < 0.001), with the highest number of sporangia produced on Bintje, then on Robijn, when the lowest number of sporangia was observed on Möwe (Table 2). In the second experiment, SS was significantly impacted by temperature (ANOVA, *F*_2,91_ = 54.804, *P* < 0.001): the lower the temperature, the bigger the sporangia (Table 2). In this experiment, temperature also had a significant effect on SN (ANOVA, *F*_2,91_ = 106.31, *P* < 0.001) with in this case, an increase of SN with increasing temperatures (Table 2). In experiment 3, no significant effect of isolate origin was detected on SS [*t*(40) = 1.63, *P* = 0.111; Table 2], nor on SN [*t*(40) = 1.81, *P* = 0.079; Table 2].

**Table 2:** Mean values (SE) of sporangia size and sporangia number measured in the three experiments for each tested factor (potato cultivar, temperature, and host of origin).

		Sporangia size (μm^3^)			Sporangia number (No. sporangia/leaflet)		
Exp. 1	Bintje	8871	(138)	a	3.01 × 10^5^	(10591)	a
	Möwe	8814	(108)	ab	1.10 × 10^5^	(4984)	c
	Robijn	8477	(85)	b	1.64 × 10^5^	(6753)	b
Exp. 2	10°C	7265	(204)	a	9.07 × 10^4^	(6259)	c
	14°C	6169	(143)	b	2.10 × 10^5^	(12247)	b
	18°C	4965	(91)	c	3.89 × 10^5^	(8768)	a
Exp. 3	Potato	8650	(275)	a	1.32 × 10^5^	(18493)	a
	Tomato	8011	(430)	a	9.56 × 10^4^	(17792)	a


*Different letters in a column indicates significant differences between means within an experiment at a 0.05 threshold (Tukey HSD or Student-t-tests).*

### Relationship Between Life-History Traits

**FIGURE 1:**
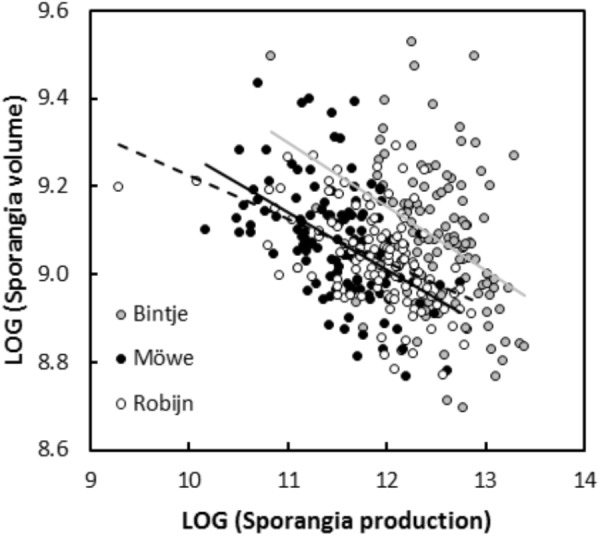
Relationship between size and number of sporangia produced by 119 *P. infestans* isolates after inoculations on potato cultivars Bintje, Möwe, and Robijn (experiment 1). All variables were LOG-transformed. For sporangia produced on Bintje, the linear regression is represented by a continuous gray line (*y* = 10.89–0.14×, *R*^2^ = 0.13, *P* < 0.001), for sporangia produced on Möwe, the linear regression is represented by a continuous black line (*y* = 10.58–0.13×, *R*^2^ = 0.23, *P* < 0.001), and for isolates inoculated on Robijn, the linear regression is represented by a dotted black line (*y* = 10.22–0.10×, *R*^2^ = 0.24, *P* < 0.001).

**FIGURE 2:**
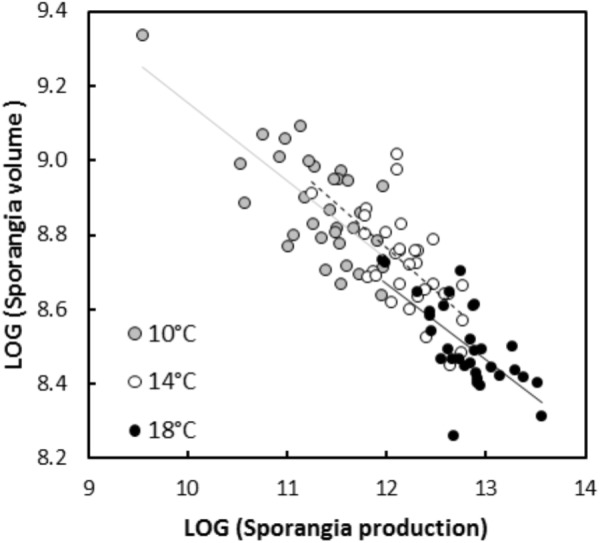
Relationship between size and number of sporangia produced by 16 *P. infestans* isolates after inoculations on potato cultivar Bintje at 10, 14, and 18°C (experiment 2). All variables were LOG-transformed. For sporangia produced at 10°C, the linear regression is represented by a continuous gray line (*y* = 11.25–0.21×, *R*^2^ = 0.49, *P* < 0.001), for sporangia produced at 14°C, the linear regression is represented by a dotted black line (*y* = 11.58–0.23×, *R*^2^ = 0.39, *P* < 0.001) and for isolates inoculated at 18°C, the linear regression is represented by a continuous black line (*y* = 11.14–0.21×, *R*^2^ = 0.46, *P* < 0.001).

**FIGURE 3:**
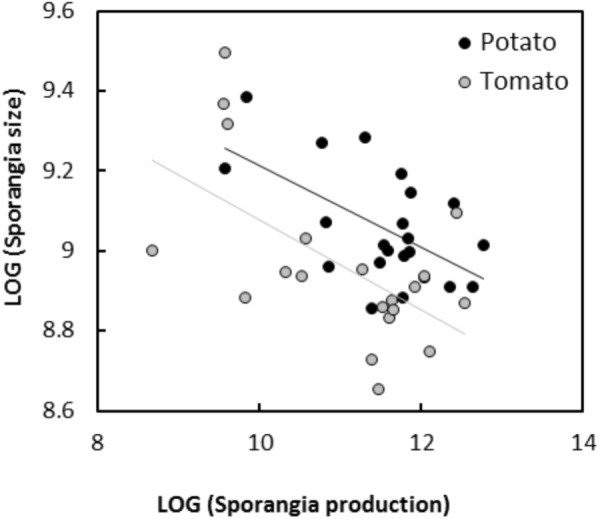
Relationship between size and number of sporangia produced by 21 *P. infestans* isolates sampled on potato or tomato after inoculations on potato cultivar Bintje (experiment 3). All variables were LOG-transformed. For sporangia produced by potato isolates, the linear regression is represented by a continuous black line (*y* = 10.23–0.10×, *R*^2^ = 0.32, *P* = 0.006) and for sporangia produced by tomato isolates, the linear regression is represented by a continuous gray line (*y* = 10.20–0.11×, *R*^2^ = 0.34, *P* = 0.007).

In experiment 1, significant negative relationships between SS and SN of *P. infestans* isolates were detected in the tests conducted on potato cultivars Bintje (*r* = -0.36, *P* < 0.001; Figure 1), Robijn (*r* = -0.49, *P* < 0.001; Figure 1), and Möwe (*r* = -0.48, *P* < 0.001; Figure 1). ANCOVA revealed no significant interaction between host cultivar and SN (Table 3), indicating that the relationship between SS and SN did not significantly differ among potato cultivars. Likewise, in the second experiment, the relationship between SS and SN was significantly negative at 10°C (*r* = -0.70, *P* < 0.001; Figure 2), 14°C (*r* = -0.62, *P* < 0.001; Figure 2), and 18°C (*r* = -0.68, *P* < 0.001; Figure 2). Again, this relationship did not significantly differ among temperatures, since no significant interaction between temperature and SN was detected by ANCOVA (Table 3). Finally, in experiment 3, SS and SN were also negatively correlated and this relationship was significant for isolates sampled on both potato (*r* = -0.57, *P* = 0.006; Figure 3) and tomato (*r* = -0.58, *P* = 0.007; Figure 3). As in the other two experiments, no significant interaction was revealed by ANCOVA between ‘origin’ and SN (Table 3), indicating that the SS-SN relationship was not significantly different between tomato and potato isolates.

**Table 3:** Summary of analyze of covariance (ANCOVA) for testing the effects of biotic and abiotic factors on the relationship between size and number of sporangia produced by *P. infestans* isolates.

Source of variation	*df*	Mean Sq	*F*
**Experiment 1**			
SN	1	1.19	77.730***
Cultivar	2	0.35	22.856***
SN × Cultivar	2	0.02	1.003
Error	351	0.02	
**Experiment 2**			
SN	1	0.72	72.610***
Temperature	2	0.05	4.854**
SN × Temperature	2	0.00	0.099
Error	90	0.01	
**Experiment 3**			
SN	1	0.43	19.036***
Origin	1	0.21	9.419**
SN × origin	1	0.00	0.044
Error	38	0.02	


*In the models, potato cultivar (experiment 1), temperature (experiment 2), and host of origin (experiment 3) were rated as factors, sporangia production (SP) as the covariate and sporangia size as the response variable. Statistical significances are indicated as follow (^∗∗^P < 0.01 and ^∗∗∗^P < 0.001). Sporangia size and sporangia number were LOG-transformed.*

## Discussion

The trade-off between offspring number and offspring size has been intensely studied and well characterized in animals and plant species (Werner and Platt, 1976; Charnov and Ernest, 2006; Walker et al., 2008). Here, we report that it also applies to a microorganism pathogenic to plants, potato late blight oomycete pathogen *P. infestans*: higher offspring numbers (in this case, asexual sporangia) correlates in this species with smaller offspring size. This trade-off has been seldom investigated in plant pathogens, even though a similar finding was reported in another plant pathogenic oomycete, the grapevine downy mildew pathogen *Plasmopara viticola* (Delmas et al., 2014). Such a trade-off is thus probably present in other sporulating parasites.

Interestingly, we have found significantly negative relationships between SS and number for all the modalities tested in our study, and with similar slopes within each experiment. Since host cultivar, temperature and isolate origin (potato or tomato) are important factors for the fitness of an almost obligate pathogen such as *P. infestans* (Andrivon et al., 2013), we could have supposed that these biotic and abiotic environmental variables could have altered the shape of the trade-off. Indeed, previous studies focused on plant pathogens reported for instance that host could modulate trade-offs, like those implying infectivity and transmission (Susi and Laine, 2013) or virulence and lesion size (Huang et al., 2010). Therefore, the stability of the trade-off relationship between SS and SN in *P. infestans* across a range of biotic and abiotic environments suggest that this trade-off is constitutive in this species, and might be exploited in control strategies of late blight epidemics.

The stability of the trade-off relationship over the range of environments tested is all the more remarkable that the environmental factors investigated strongly impacted one or both of the life history traits involved in the trade off. In fact, we showed that sporangia production of *P. infestans* was strongly host-dependent, confirming earlier observations made in this species (Clément et al., 2010) and more generally in a range of fungal and oomycete parasites (Hardham and Hyde, 1997; Delmotte et al., 2014). Interestingly, we also showed a clear effect of potato cultivars on SS, with smaller sporangia on Robijn – especially compared to Bintje. A similar observation was reported in *P. viticola* (Delmotte et al., 2014) and *Venturia inaequalis* (De Gracia et al., 2015), suggesting a possible selection by host on this trait among sporulating plant pathogens. Regarding temperature, the thermal optimum for *P. infestans* development is around 18–20°C (Mizubuti and Fry, 1998; Mariette et al., 2016), and spore production quickly decreases beyond this. In our second experiment, sporangia were much less numerous at the lowest temperatures, but they were bigger. This phenomenon could be due to the longer latent period of the pathogen at low temperatures (Maziero et al., 2009; Mariette et al., 2016), leading to more time for sporangia formation. Finally, we observed that isolates sampled on tomato produced less sporangia on the potato cultivar Bintje than those sampled on potato, confirming the host specialization previously highlighted in *P. infestans* (Oyarzun et al., 1998; Kröner et al., 2017).

By preventing an organism to simultaneously maximize all traits involved in fitness, trade-offs can equalize fitness across individuals, genotypes, or species with different reproductive strategies, leading to the maintenance of phenotypic diversity (Roff, 1992). For instance, Héraudet et al. (2008) suggested that the trade-off between latent period and spore production in *H. arabidopsidis* may explain why the latent period remains variable in nature and did not achieve a uniformly minimal value. The constraint of total reproductive effort imposing a trade-off in the size and number of sporangia in *P. infestans*, as observed in our study, could therefore maintain variability for these life-history traits within populations of this pathogens. This tends to be confirmed with the large range of sporangia production values generally reported amongst isolates of single *P. infestans* populations (e.g., Montarry et al., 2008b, 2010; Mariette et al., 2016). Moreover, the polyphenism of life-history traits values imposed by a trade-off can lead to the emergence of various life-history strategies (Roff, 1992). This can be crucial in pathogens highly dependent on their environmental variations, as different life-history strategies can explain the coexistence of genetically differentiated clades, in complete or partial reproductive isolation, within single plant pathogen species (Montarry et al., 2008a).

Here, we report that a trade-off between number and size of asexual spores occurred in an important oomycete plant pathogen, *P. infestans*, and that its shape was not affected by different biotic and abiotic factors. It would be interesting to know the relationship between asexual spore number and size in other fungal plant pathogens as trade-offs (and their shape) can play a key role in evolutionary outcomes of these organisms (Kamo et al., 2007). Nevertheless, it is far from being the case at present, because if the production of the asexual propagules (spore or sporangia) is a trait extensively studied in fungal plant pathogens (Hardham and Hyde, 1997; Montarry et al., 2010), their size is still often ignored. Yet, the size of fungal propagules may be an important trait for different aspects of their fitness. The infection efficiency should be higher for large spores as they are supposed to have more resources available for hyphal growth (Bässler et al., 2015). Likewise, SS in oomycetes could also impact their indirect germination (formation of zoospores) as suggested by investigations on *P. viticola* showing that larger sporangia produced more zoospores (Delmas et al., 2014). Conversely, small spores might have higher aerial dispersal abilities and could therefore tend to deposit further to the source than larger ones (Norros et al., 2014). Nevertheless, owing to the lack of empirical evidences, the link between asexual spore size and such fitness aspects remain uncertain (De Gracia et al., 2015). Further research efforts on this topic are thus needed and it would be particularly crucial to study the impact of offspring size on virulence or disease lesion development in fungal plant pathogens. Besides, a future way of research in plant pathogens could be to not only limit on the trade-off between number and size of offspring but, as recommended by Begon et al. (2006), to look for a trade-off between the number of offspring and their individual fitness.

## Data Availability Statement

The datasets generated for this study will be found on data.inra.fr (doi: 10.15454/BB0WJN).

## Author Contributions

RM, AA, BM, NM, and AK performed the experiments according to a protocol elaborated jointly by RC and DA. NM and AK analyzed the data. NM, JM, RC, and DA wrote the text and prepared the figures. All authors edited the paper and have approved the current version.

## Conflict of Interest Statement

The authors declare that the research was conducted in the absence of any commercial or financial relationships that could be construed as a potential conflict of interest.
